# New Insights via RNA Profiling of Formalin-Fixed Paraffin-Embedded Lung Tissue of Pulmonary Fibrosis Patients

**DOI:** 10.3390/ijms242316748

**Published:** 2023-11-25

**Authors:** Dymph Klay, Karin M. Kazemier, Joanne J. van der Vis, Hidde M. Smits, Jan C. Grutters, Coline H. M. van Moorsel

**Affiliations:** 1 Interstitial Lung Diseases Center of Excellence, Department of Pulmonology, St. Antonius Hospital, 3435 CM Nieuwegein, The Netherlands; 2 Center of Translational Immunology, University Medical Center Utrecht, 3584 CX Utrecht, The Netherlands; 3 Division of Heart and Lungs, University Medical Center Utrecht, 3584 CX Utrecht, The Netherlands; 4 Department of Clinical Chemistry, ILD Center of Excellence, St. Antonius Hospital, 3435 CM Nieuwegein, The Netherlands

**Keywords:** idiopathic pulmonary fibrosis, surfactant related gene mutation, telomere related gene mutation, SFTPC, SFTPA2, RTEL1, TERT, lung tissue, RNA expression

## Abstract

In sporadic idiopathic pulmonary fibrosis (sIPF) and pulmonary fibrosis caused by a mutation in telomere (TRG-PF) or surfactant related genes (SRG-PF), there are a number of aberrant cellular processes known that can lead to fibrogenesis. We investigated whether RNA expression of genes involved in these processes differed between sIPF, TRG-PF, and SRG-PF and whether expression levels were associated with survival. RNA expression of 28 genes was measured in lung biopsies of 26 sIPF, 17 TRG-PF, and 6 SRG-PF patients. Significant differences in RNA expression of *TGFBR2* (*p* = 0.02) and *SFTPA2* (*p* = 0.02) were found between sIPF, TRG-PF, and SRG-PF. Patients with low (<median) expression of *HSPA5* (*p* = 0.04), *COL1A1* (*p* = 0.03), and *ATF4* (0.005) had significantly longer survival rates than patients with high (≥median) expression of these genes. In addition, we scored for low (0) or high (1) expression of six endoplasmic reticulum (ER) stress genes (*HSP90B1*, *DDIT3*, *EDEM1*, *HSPA5*, *ATF4,* and *XBP1*) and found that patients with high expression in a low number of ER stress genes (total score 0–1) had longer survival rates than patients with high expression in a high number of ER stress genes (total score 2–6) (*p* = 0.03). In conclusion, there are minor differences between sIPF, TRG-PF, and SRG-PF and high expression in a high number of ER stress genes significantly associated with shorter survival time, suggesting that ER stress may be a target for therapy for PF.

## 1. Introduction

Idiopathic pulmonary fibrosis (IPF) is characterized by damage to the alveolar epithelium and accumulation of extracellular matrix in the interstitium. IPF has a poor prognosis with a median survival of approximately 3–4 years [1,2]. The disease is highly heterogeneous, while in the majority of patients etiology is unknown, some of the patients have genetic pulmonary fibrosis. Pathogenic mutations causing pulmonary fibrosis have been identified in two different groups of genes, surfactant related genes (SRG) such as *SFTPC* and *SFTPA2*, or telomere related genes (TRG) such as *TERT* and *RTEL1* [3]. In a study by Snetselaar et al. [4], telomere length was measured in white blood cells of patients with sporadic IPF (sIPF) and SRG-PF and TRG-PF. It was shown that some sIPF patients had short telomeres comparable with TRG-PF, whereas others had a telomere length comparable with SRG-PF. This is in congruence with a recent report showing the wide range in telomere length in patients with pulmonary fibrosis who do not carry a likely pathogenic mutation [5]. This suggests that disease drivers in sIPF may overlap with those in specific genetic groups.

In previous studies, several molecular processes involved in IPF have been identified such as extracellular matrix deposition [6,7], endoplasmic reticulum (ER) stress [8,9], senescence [10,11], and hypoxia [12]. In addition, different cell types of the bronchoalveolar compartment, and particularly the alveolar type 2 cell (AT2), were found to be involved in the pathogenesis of IPF [13]. IPF expression studies showed an increase in expression of fibrogenesis related genes such as *TGFB1*, *ACTA2* [14,15,16], and hypoxia genes including *HIF1A* and *EPAS1* [12,17,18], but also involvement of cellular processes such as autophagy [19,20]. Mutations in surfactant and telomere genes were shown to each lead to different processes in the lung, and it is unknown to what extent these processes overlap. Heterozygous mutations in SRG have a toxic gain of function effect on surfactant processing [21,22,23] and may cause ER stress with upregulation of ER stress associated genes, such as *HSPA5* and *XBP1* [24,25,26]. On the other hand, heterozygous TRG mutations cause haploinsufficiency leading to excessive shortening of telomeres and were shown to increase DNA damage related processes including upregulation of *TP53BP1* and *TP53* [27] and cause senescence with altered expression of *CDKN2A* and *CDKN1A* [28]. Because disease cause and outcome are so heterogeneous in PF, a better understanding of the involvement of the different processes and their relation to patient survival is warranted and may aid development of therapies targeting specific processes in patients. To determine whether TRG-PF and SRG-PF have distinct expressions of genes involved in disease pathogenesis, we measured RNA expression in diagnostic lung biopsies of sIPF, TRG-PF, and SRG-PF. In addition, we investigated whether different levels of RNA expression are associated with survival.

## 2. Results

**Figure 1:**
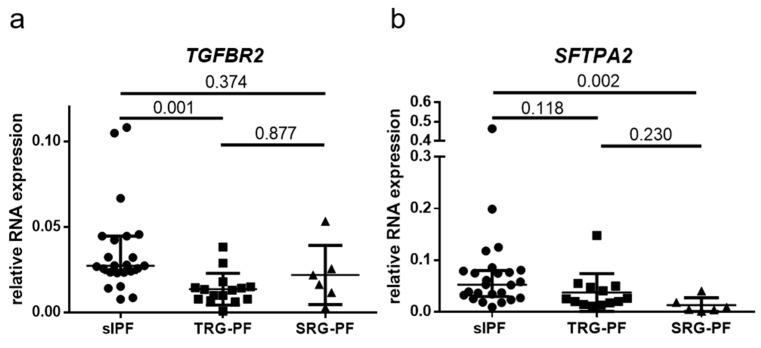
Relative RNA expression in lung tissue of sporadic IPF (sIPF) patients and pulmonary fibrosis patients with a telomere related gene (TRG-PF) or surfactant related gene (SRG-PF) mutation (**a**). Relative RNA expression of *TGFBR2*. A significant difference in RNA expression was found between the three groups (Kruskal–Wallis test *p* = 0.02). Post hoc analysis showed significantly higher expression in sIPF compared to TRG-PF (*p* = 0.001). There were no significant differences in expression between sIPF and SRG-PF (0.374) or between TRG-PF and SRG-PF (0.877). (**b**). Relative RNA expression of *SFTPA2*. A significant difference in RNA expression was found between the three groups (Kruskal–Wallis test *p* = 0.02). Post hoc analysis showed significantly higher expression in sIPF compared to SRG-PF patients (*p* = 0.002). There were no significant differences in expression between sIPF and TRG-PF (0.118) or between TRG-PF and SRG-PF (0.23).

RNA expression of 28 genes involved in IPF pathogenesis was measured in FFPE surgical lung biopsies of three groups of patients. (26 sIPF, 17 TRG-PF, and 6 SRG-PF). Clinical characteristics of the three patient groups are displayed in Table 1. Telomere length in tissue and blood was significantly different between the three groups (*p* = 0.001 and *p* < 0.001, respectively). Telomere length was lower in TRG-PF patients than the other two patient groups. In addition, male predominance was present in sIPF (92.3%) and TRG-PF patients (82.4%), but not in SRG-PF patients (50%). The 28 genes are associated with different processes, such as senescence, DNA damage, endoplasmic reticulum (ER) stress, surfactant homeostasis, extracellular matrix (ECM), autophagy, hypoxia, and protein degradation. Statistical analysis showed no difference in RNA expression of 25 genes (expression of *TP53*, *CDKN2A,* and *CDKN1A* was not measured in SRG-PF patients) between TRG-PF and SRG-PF patients. Comparison of RNA expression of the 28 genes between sIPF, TRG-PF, and SRG-PF showed a significant difference in RNA expression of *TGFBR2* (*p* = 0.02) and *SFTPA2* (*p* = 0.02). Post-hoc analysis showed significantly lower expression of *TGFBR2* in TRG-PF compared to sIPF patients (*p* = 0.001) and lower expression of *SFTPA2* in SRG-PF compared to sIPF patients (*p* = 0.002, Figure 1). Two out of three patients with an *SFTPC* mutation had the lowest levels of *SFTPC* expression. The median *SFTPC* expression in the *SFTPC* mutation carriers was just 0.03. This was not significantly different from the *SFPTC* level of 0.05 in the patients carrying an SFTPA2 mutation. Similarly, two out of three patients carrying an *SFTPA2* mutation had the lowest SFTPA2 expression and the median *SFTPA2* expression in the entire *SFTPA2* group was just 0.008. However, this was not significantly lower than the *SFTPA2* expression in the patients carrying an *SFTPC* mutation (0.004).

### 2.1. Clustering of Genes and Patients

**Figure 2:**
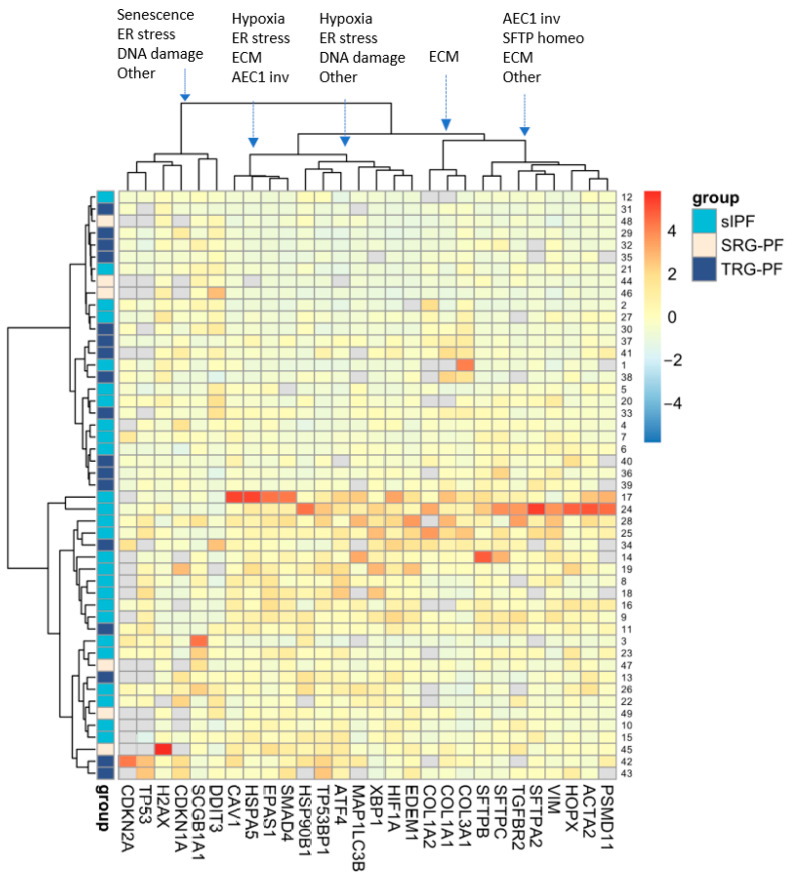
Unsupervised two-way hierarchical clustering of RNA expression of 28 genes in 49 patients. Rows represent individual patients and columns represent genes. Grey blocks indicate that RNA expression of a certain gene was not measured due to shortage of tissue or undetectable expression. Clustering of process related genes in each of the five main gene clusters is indicated at the top with arrows pointing to the cluster. AEC1 inv: alveolar epithelial type 1 involvement, SFTP homeo: surfactant homeostasis; ECM: extracellular matrix; other: this includes bronchiolar involvement, autophagy, or protein degradation.

Unsupervised two-way hierarchical clustering of RNA expression of the 28 genes in 49 patients demonstrated five major vertical clusters of genes and two major horizontal clusters of patients (Figure 2). The upper cluster of patients contains 10 sIPF, 12 TRG-PF, and 3 SRG-PF patients, whereas the lower cluster contains 16 sIPF, 5 TRG-PF, and 3 SRG-PF patients. The distribution of patient groups was not significantly different between the two clusters (*p* = 0.12). Analysis of the clinical characteristics of the two clusters showed no differences except for a significantly higher number of deaths in the lower cluster compared to the upper cluster (*p* = 0.04, Table 2).

The 28 measured genes can be categorized in gene process groups: surfactant homeostasis (*SFTPC*, *SFTPA2*, and *SFTPB*), ER-stress (*HSP90B1*, *EDEM1*, *DDIT3*, *ATF4*, *XBP1*, and *HSPA5*), DNA-damage (*TP53BP1*, *H2AX*, and *TP53*), extracellular matrix (*ACTA2*, *VIM*, *COL1A1*, *COL1A2*, *COL3A1*, *SMAD4*, and *TGFBR2*), hypoxia (*HIF1A* and *EPAS1)*, senescence (*CDKN2A* and *CDKN1A*), protein degradation (*PSMD11*) and autophagy (*MAP1LC3B*), and AEC1 involvement (*HOPX* and *CAV1*) and bronchiolar involvement (*SCGB1A1*). *HOPX* and *CAV1* encode proteins regulating the repair of AEC1 after injury. *SCGB1A1* encodes a secretoglobin that exerts an anti-inflammatory function in the small airways. Genes were not randomly distributed over the five major vertical clusters (Figure 2). Both senescence genes were part of one cluster, and all other three surfactant homeostasis genes were part of another cluster. Furthermore, the three collagen encoding genes formed a separate cluster.

### 2.2. Survival

**Figure 3:**
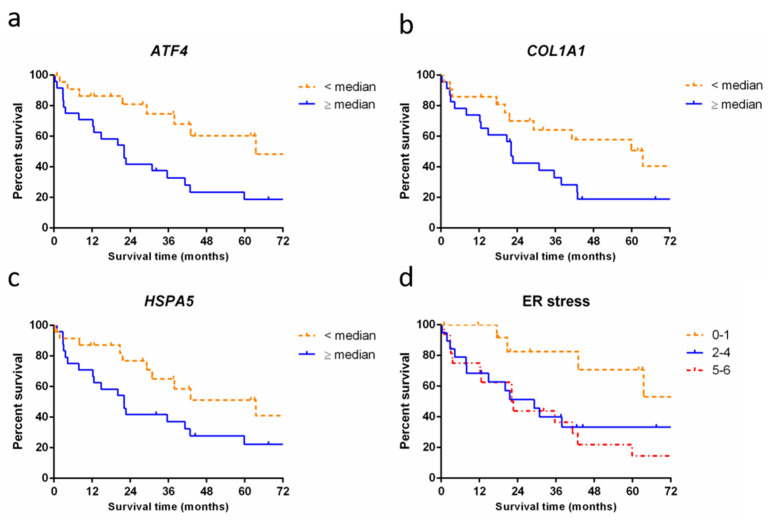
Survival of patients with pulmonary fibrosis stratified by RNA expression levels. (**a**) Survival curve showing significantly longer survival in patients with low expression of ATF4 (64 months, *n* = 23, dashed line) compared to patients with high expression of ATF4 (22 months, *n* = 24, solid line, *p* = 0.005); (**b**) Survival curve showing a significantly longer survival in patients with low expression of COL1A1 (64 months, *n* = 22, dashed line) compared to patients with high expression of COL1A1 (22 months, *n* = 23, solid line, *p* = 0.03). (**c**) Survival curve showing a significantly longer survival in patients with low RNA expression of HSPA5 (64 months, *n* = 24, dashed line) compared to patients with high expression of HSPA5 (22 months, *n* = 24, solid line, *p* = 0.04). (**d**) Accumulative score in patients for high (+1 point) or low (0 points) expression in each of six ER stress genes (*HSP90B1*, *DDIT3*, *EDEM1*, *HSPA5*, *ATF4,* and *XBP1*). Survival curve showing significantly longer median survival in patients with high RNA expression of 0–1 ER stress genes (95 months, *n* = 14, dashed line) compared to patients with high expression of 2–4 ER stress genes (29 months, *n* = 19, solid line) and high expression of 5–6 ER stress genes (22 months, *n* = 16, dash-dotted line, *p* = 0.03).

We investigated whether overall survival was different between patients with high and low RNA expression of a gene. Comparison showed significantly longer survival in patients with low (<median) RNA expression of ER stress genes *ATF4* (64 months vs. 22 months; *p* = 0.005), *HSPA5* (64 months vs. 22 months; *p* = 0.04), and for ECM gene *COL1A1* (64 months vs. 22 months; *p* = 0.03) compared to patients with high (≥median) RNA expression of these genes (Figure 3a–c).

Next, we grouped three or more genes that play a role in the same processes into process expression groups: surfactant homeostasis (*SFTPB*, *SFTPC,* and *SFTPA2*), DNA damage (*TP53BP1*, *H2AX*, and *TP53*), ECM (*ACTA2*, *VIM*, *TGFBR2*, *COL1A1*, and *SMAD4*), and ER stress (*HSP90B1*, *DDIT3*, *EDEM1*, *HSPA5*, *ATF4,* and *XBP1*). For each patient, a process expression score was calculated based on the sum of above (+1) or below (0) median expression for each gene in the process. Comparison of overall survival between high, average, and low scoring patients showed a significant difference for ER stress: patients with low expression of ER stress genes (score 0–1) showed a longer survival time than patients with average or high expression of these genes (score 2–4 or 5–6). Median survival in low expressing ER stress patients was 95 months versus 29 and 22 months in the average and high expressing patients, respectively (*p* = 0.03, Figure 3d). Survival analysis results of surfactant homeostasis, DNA damage, and extracellular matrix showed no significant differences (see Supplementary Materials). Results for processes represented by only two genes were also included in the Supplementary Materials.

## 3. Discussion

Several processes have been associated with the development of PF in general and with surfactant or telomere related PF specifically. In this study, we quantified RNA expression of proteins that have previously been shown to be involved in pulmonary fibrosis in lung biopsies of patients with sporadic and genetic IPF and analyzed the effect on survival. There was no significant clustering of patient groups and genes and no significant differences in RNA expression between the groups with TRG-PF and SRG-PF. This is the first study that showed significantly worse survival in PF patients with high expression of two or more ER stress genes compared to PF patients with low expression of five or all six ER stress genes.

Several gene expression studies have been performed on lung tissue from patients with pulmonary fibrosis. First, it was shown that expression in IPF patients differed significantly from control subjects. In a study by Wang et al. [29], microarray data from lung tissue of 131 IPF patients with UIP on pathology and 12 controls were used. Comparison of IPF patients and controls resulted in 988 differentially expressed genes. In addition, by ward clustering and principal component analysis of gene expression profiles of 131 IPF patients, six patient clusters were found. The six clusters differed in disease severity and differential expression compared to controls in genes that play a role in processes such as extracellular matrix organization, regulation of cell migration, collagen catabolic process, cilium, cilium assembly, angiogenesis, and lung alveolar morphology. Furthermore, in a whole genome oligonucleotide microarray study by Yang et al. [30], RNA expression of 41,000 genes and transcripts was measured in lung tissue of 16 sporadic idiopathic interstitial pneumonia patients, 10 familial idiopathic interstitial pneumonia patients, and 9 normal control subjects. In total, 135 transcripts were found to be upregulated or downregulated more than 1.8-fold in pulmonary fibrosis patients compared to normal control subjects. After hierarchical clustering, four patient clusters were identified; all controls except for two samples clustered together and all familial idiopathic interstitial pneumonia except for three samples clustered together. Comparison between sporadic idiopathic interstitial pneumonia (IIP) and familial IIP patients resulted in 142 transcripts from 62 genes with known functions that were more than 1.8-fold upregulated or downregulated. Interestingly, they found that genes from the same functional categories, such as calcium/potassium binding, cell adhesion, cell proliferation and death, ECM degradation, and cytokines/chemokines, are differentially expressed between sporadic and familial IIP as well as between IIP and normal control subjects, but then to a larger extent in the familial IIP patients. But because no genetic analysis was performed, it remains unclear whether patients carrying a TRG or SRG mutation had been included.

In our study, we included patients with familial disease of known cause and divided them over two groups: patients carrying an SRG and patients carrying a TRG mutation and studied expression of genes involved in mutation driven aberrant processes in PF. To our surprise, we detected no differences in expression of any of the studied genes between the TRG and SRG-PF groups. However, when we compared sIPF, TRG-PF, and SRG-PF, we found significant differences in RNA expression of *TGFBR2* and *SFTPA2* between the three groups. The cluster analysis did not result in clustering of patients in their respective sIPF, TRG-PF, or SRG-PF patient group. On the other hand, there was clustering of genes belonging to the same processes. As surfactant homeostasis genes clustered together and senescence genes also clustered together, this indicates that the expression levels may correctly inform about the activity of the process.

Lawson et al. studied protein expression in AECs in lung tissue of three patients with pulmonary fibrosis and an *SFTPC* mutation, ten patients with familial interstitial pneumonia without an *SFTPC* mutation, and ten patients with sIPF. They observed expression of the ER stress proteins BiP, EDEM, and XBP1 in lung tissues of all patients [8]. In addition, Carleo et al. investigated protein patterns in bronchoalveolar lavage (BAL) fluid of 10 familial and 17 sporadic IPF patients. In total, 22 proteins were found to be differentially expressed between familial and sporadic IPF. The upregulated proteins in sIPF played a role in oxidative stress response and the upregulated proteins in familial IPF (including SP-A2 encoded by *SFTPA2*) played a role in immune response, coagulation system, wounding response, and ion homeostasis [31]. The increase of SP-A2 in BAL fluid of familial IPF patients in Carleo et al. is in contrast with the lower RNA expression of *SFTPA2* in lung tissue of the SRG-PF patients in our study. Although their small familial cohort was not analyzed for carriage of genetic mutations, it is unlikely that their familial cohort included any patients with an SRG mutation. SRG mutations occur in 3–8% of patients with familial disease, while TRG mutations are present in approximately 35% of familial patients [3]. However, we did not observe a difference in *SFTPA2* RNA expression between our sIPF and familial TRG-PF patients. The level of RNA expression may therefore not reflect protein expression, while increased SP-A2 in BAL fluid may aid identification of familial patients, and the lower RNA expression of *SFTPA2* in lung tissue may aid identification of patients with surfactant-related pathology.

Furthermore, the SP-A2 protein level in blood was shown to be a predictive marker for progressive disease [32,33]. Although it remained unclear what the source is of SP-A2 in blood, and how increased levels in blood mechanistically related to progression of disease in the lung. When analyzing the survival of patients, TRG-PF showed the shortest survival (Table 1), but the survival of patients with SRG-PF could not be calculated due to the low number. However, in a recent study, survival of SRG-PF patients was shown to be comparable with that of sIPF [34]. Our study suggests that in patients with SRG-PF, levels may be influenced by the presence of surfactant related mutations, and thus future investigations into biomarkers for PF may benefit from stratification of patients by genetic cause of disease.

When we studied the impact of RNA expression of each gene on survival in patients, we found that patients with low expression of *HSPA5*, *COL1A1,* or *ATF4* showed a significantly longer survival rate than patients with high expression of one of these genes. In a study by Tsitoura et al. [35], they observed increased *COL1A1* RNA expression in BAL cells of patients with 53 IPF and 62 non-IPF ILD compared to 19 controls. In addition, they also found that high levels of *COL1A1* expression were associated with worse survival. However, this was only found in the non-IPF ILD patients and not in the IPF cohort. For ER stress gene *ATF4*, as far as we know, no association with survival was investigated before in other studies, although it was found that ATF4 expression was increased in IPF lung tissue and colocalized with apoptosis markers CHOP and cleaved caspase 3, and encoded by *DDIT3* and *CASP3,* respectively, in alveolar epithelial cells overlying fibroblast foci [9]. For ER stress marker GRP78/BiP encoded by *HSPA5,* conflicting results have been found; in one study elevated GRP94 and CHOP, encoded by *HSP90B1* and *DDIT3,* respectively, and reduced expression of GRP78 were observed in alveolar epithelial cells type 2 (AEC2s) from IPF lung tissue compared to AEC2s from normal donors [36], whereas in another study increased GRP78/BiP expression was observed in IPF lung tissue compared to control lung tissue. In the latter study, strong GRP78/BiP staining was found in alveolar epithelial cells in regions of fibroblasts foci [37]. Fibroblasts foci are aggregates of proliferating fibroblasts and myofibroblasts thought to represent areas of active fibrosis. Therefore, the proximity of strong BiP staining (encoded by *HSPA5*) to fibroblast foci suggests a role in fibrogenesis. Together with our finding that high expression of ER stress genes, including *ATF4* and *HSPA5*, results in low survival, the evidence suggests that ER stress in IPF may be an important factor for progression of fibrogenesis. It is known that the cause of ER stress may be different in each patient [38]. However, the absence of differences in expression between our groups of patients and the presence of a correlation with survival suggest that ER stress is an important factor in the progression of pulmonary fibrosis independent of underlying genetic cause. Two recent studies show the feasibility of targeting ER stress in pulmonary fibrosis. Kropski et al. [39] showed that ER stress may be targeted by pharmacological chaperones such as sodium phenylbutyrate, which resulted in reduced ER stress in preclinical disease models or by drugs that selectively inhibit oligomerized IRE1α, and which have been investigated in animal models. Chen et al. [40] showed that inhibition of IRE1α by an endoribonuclease inhibitor alleviates CS-induced pulmonary inflammation and fibrogenesis in a mouse model.

A limitation of our study is that only bulk RNA expression was measured. Differences in RNA expression between cell types, such as in single cell RNA sequencing, were not investigated. In addition, no spatial transcriptomics and immunohistochemistry was used to localize which cell populations were involved in the regulation of RNA expression in PF. Furthermore, the results were not verified at the protein level and therefore effects of posttranscriptional regulation of gene expression during fibrogenesis was not investigated. The number of patients, especially those with SRG-PF, was quite low. However, the low number of SRG-PF cases was limited by the rarity of these patients compared to those with sIPF. Inclusions of more cases might have shown additional significant differences in the clinical characteristics between the two clusters. In addition, the number of genes measured may be too small to create distinct clusters. The low number of differences in RNA expression between sIPF, TRG-PF, and SRG-PF may also explain why based on most recent finding these patients appear to benefit from the same treatment [32,41,42]. In addition, although all samples were diagnostic lung biopsies, a considerably low DLCO % predicted in especially TRG-PF and SRG-PF patients was present, which suggests already advanced lung disease. It remains possible that more differences, particularly mutation associated differences, would be present if early disease samples had been analyzed. While screening family members at risk may aid early disease detection, genetic analysis is likely to reduce the need for biopsies. In a recent survey, 72% of pulmonologists reported that they might modify their diagnostic work-up according to the results of genetic testing of whom 78% would postpone or exclude surgical lung biopsy [43].

In conclusion, analysis of expression of fibrosis related genes in sIPF and TRG-PF and SRG-PF patients showed almost similar gene expression between the groups. The finding that high expression of ER stress genes leads to worse survival time supports development of therapies targeting ER stress that may be beneficial to all PF patients, including those with genetic pulmonary fibrosis. Further studies are needed to investigate this association in more detail.

## 4. Materials and Methods

### 4.1. Patients and Tissue Selection

Diagnostic surgical lung biopsies and blood obtained between 1997 and 2016 of 26 sIPF, 17 TRG-PF (*RTEL n* = 3, *TERT n* = 14), and 6 SRG-PF (*SFTPC n* = 3, *SFTPA2 n* = 3) patients were included in this study. Diagnoses were based on the ATS/ERS/JRS/ALAT guidelines [44]. All subjects signed written consent for the study approved by the Medical Research Ethics Committees United (MEC-U) of the St. Antonius hospital (R05-08A).

### 4.2. DNA/RNA Isolation from Lung Tissue and Blood

After removal of paraffin with paraffin dissolver (Macherey-Nagel, Düren, Germany), RNA and DNA was isolated from formalin-fixed paraffin embedded tissue using AllPrep DNA/RNA FFPE kit (Qiagen Benelux BV, Venlo, The Netherlands). DNA and RNA concentration and purity were measured using a NanoDrop spectrophotometer. For isolation of genomic DNA from peripheral white blood cells, a magnetic beads-based method (chemagic DNA blood 10k kit; Perkin Elmer Inc., Waltham, MA, USA) was used.

### 4.3. Telomere Length Measurements in Lung Tissue and Blood

T/S ratio was measured in isolated DNA from tissue and blood by monochrome multiplex quantitative polymerase chain reaction (MMqPCR) as described before in [45,46,47]. The T/S ratio is a measure for telomere length. Telomere length adjusted for age was calculated by the difference between the observed T/S ratio and the age-adjusted normal value (T/S expected).

### 4.4. Real-Time PCR

cDNA (6ng per reaction), prepared from RNA using i-script (Bio-Rad GmbH laboratories B.V), was amplified using iQ SYBR Green Supermix (Bio-Rad GmbH laboratories B.V, Lunteren, The Netherlands) and gene specific primers (see Supplementary Materials) in a CFX96 Bio-Rad qPCR machine with the following run conditions: 3 min at 95 °C followed by 45 cycles of 10 s at 95 °C, 20 s at 61 °C, and 25 s at 72 °C. RNA expression was calculated by delta Ct method using the mean of three reference genes: *ACTB*, *RPL13A,* and *EEF1A1*. RNA expression was investigated for genes involved in the following processes: bronchiolar involvement (*SCGB1A1*), alveolar epithelial cell type 1 (AEC1) involvement (*HOPX* and *CAV1*), surfactant homeostasis (*SFTPC*, *SFTPA2*, *SFTPB*), hypoxia (*HIF1A*, *EPAS1)*, protein degradation (*PSMD11*), autophagy (*MAP1LC3B*), senescence (*CDKN2A*, *CDKN1A*), ER-stress (*HSP90B1*, *EDEM1*, *DDIT3*, *ATF4*, *XBP1*, *HSPA5*), DNA-damage (*TP53BP1*, *H2AX*, *TP53*) and extracellular matrix (*ACTA2*, *VIM*, *COL1A1*, *COL1A2*, *COL3A1*, *SMAD4*, *TGFBR2*). List of used primers can be found in Table S1.

### 4.5. Statistical Analysis

For analysis of differences in demographics and clinical characteristics between sIPF, TRG-PF, and SRG-PF patients, the chi-square test was used for discrete variables and the Kruskal–Wallis test was used for continuous variables, as appropriate with a small sample size. The Mann–Whitney U test was used to compare RNA expression between TRG-PF and SRG-PF followed by false discovery rate (FDR) to correct for multiple comparisons. The Kruskal–Wallis test was used to analyze differences in RNA expression between sIPF, TRG-PF, and SRG-PF followed by FDR to correct for multiple comparisons. Unsupervised two-way hierarchical clustering of RNA expression using Ward’s clustering method with Euclidean distance metric was used to create a heatmap. Chi-square and Mann–Whitney U test were used for discrete and continuous variables, respectively, to compare demographics and clinical characteristics between the two horizontal clusters in the heatmap. For survival analyses, median RNA expression was calculated for each gene and patients were divided into two groups containing patients with <median expression of that gene and patients with ≥median expression of that gene. Additionally, when three or more genes play a role in the same process, these were also analyzed together as one process-group, surfactant homeostasis (*SFTPB*, *SFTPC*, *SFTPA2*), DNA damage (*TP53BP1*, *TP53, H2AX*), ECM (ACTA2, *VIM*, *TGFBR2*, *COL1A1*, *SMAD4*), and ER stress (*HSP90B1*, *DDIT3*, *EDEM1*, *HSPA5*, *ATF4*, *XBP1*). For ECM, only one, *COL1A1*, of the three collagen genes was included in the survival analysis to prevent overrepresentation of collagen genes. For each process-group of genes, we determined the score for each patient. Per gene, RNA expression ≥median is +1 point and <median is 0 points. The sum of these scores resulted in an accumulative score per process per patient. For each process, we performed survival analysis dividing the patients into groups. Survival was compared using the Kaplan–Meier method with log-rank tests. Survival time was determined from time of lung biopsy until death. Patients were censored when lost to follow-up or when they underwent lung transplantation or at the last contact date. Statistical analyses were performed in SPSS v.26 and R v.4.2.2 (including the following packages: tidyverse version 1.3.2, ggsci v. 2.9, readxl v. 1.4.1, pheatmap v. 1.0.12, ggpubr v. 0.5.0).

## Figures and Tables

**Table 1:** Demographics and clinical characteristics of patient groups at time of biopsy.

Characteristic	sIPF	TRG-PF	SRG-PF	*p*
N	26	17	6	
Age biopsy, years, median (IQR)	62.8 (12.5)	58.5 (9.1)	41.9 (30.6)	0.01
Male, *n* (%)	24 (92.3)	14 (82.4)	3 (50)	0.04
Ever smoker, *n* (%)	19 (82.6)	14 (82.3)	2 (40.0)	0.06
FVC % predicted, median (IQR)	81.4 (25.9)	77.3 (24.7)	54.3 (31.6)	0.06
DLCO % predicted, median (IQR)	49.9 (15.2)	41.6 (9.6)	30.7 (20.1)	0.009
T/S ratio tissue, median (IQR)	0.839 (0.127)	0.772 (0.074)	0.830 (0.087)	0.001
T/S ratio blood, median (IQR)	0.795 (0.128)	0.690 (0.180)	0.901 (0.274)	<0.001
T/S ratio blood observed, expected, median (IQR)	−0.147 (0.149)	−0.271 (0.151)	−0.049 (0.229)	<0.001
Transplant event, *n* (%)	7 (26.9)	2 (11.7)	3 (50)	0.16
Deaths, *n* (%)	15 (57.7)	14 (82.4)	2 (33)	0.07
Overall survival time, mo, median (SE)	42.8 (19.1)	21.5 (9.3)	NA	0.05

FVC % predicted and DLCO % predicted values are ±12 months from time of biopsy. The T/S ratio is a measure for telomere length. IQR: interquartile range; mo: months; sIPF sporadic idiopathic pulmonary fibrosis; TRG-PF: telomere related gene mutation pulmonary fibrosis; SRG-PF: surfactant related gene mutation pulmonary fibrosis; FVC: forced vital capacity; DLCO: diffusing capacity of the lung for carbon monoxide. Smoking status: *n* is 23 sIPF, 16 TRG-PF, 5 SRG-PF. FVC %predicted *n* is 19 sIPF, 14 TRG-PF, 4 SRG-PF. DLCO % predicted: *n* is 17 sIPF, 13 TRG-PF, 5 SRG-PF.

**Table 2:** Demographics and clinical characteristics of two patient clusters.

Characteristic	Upper Cluster	Lower Cluster	*p*
N	25	24	
Group, sIPF/TRG-PF/SRG-PF	10/12/3	16/5/3	0.12
Age biopsy, years, median (IQR)	58.0 (13.5)	61.0 (13.8)	0.34
Male, *n* (%)	20 (80)	21 (87.5)	0.70
Ever smoker, *n* (%)	19 (79.2)	16 (80.0)	1.00
FVC % predicted, median (IQR)	82.6 (24.9)	71.7 (26.2)	0.40
DLCO % predicted, median (IQR)	45.2 (14.4)	46.7 (25.3)	0.91
T/S ratio tissue, median (IQR)	0.806 (0.081)	0.831 (0.127)	0.05
T/S ratio blood, median (IQR)	0.784 (0.189)	0.797 (0.141)	0.17
T/S ratio blood, observed, expected, median (IQR)	−0.161 (0.237)	−0.131 (0.134)	0.10
Transplant event, *n* (%)	8 (32.0)	4 (16.7)	0.32
Deaths, *n* (%)	12 (48.0)	19 (79.2)	0.04
Overall survival time, median (SE), mo	43.0 (17.8)	22.6 (6.8)	0.08

FVC % predicted and DLCO % predicted values are ±12 months of biopsy date; IQR: interquartile range; mo: months; *n*: number; sIPF sporadic idiopathic pulmonary fibrosis; TRG-PF: telomere related gene mutation pulmonary fibrosis; SRG-PF: surfactant related gene mutation pulmonary fibrosis; FVC: forced vital capacity; DLCO: diffusing capacity of the lung for carbon monoxide. Smoking status: *n* is 24 in upper cluster, 20 in lower cluster. FVC % predicted *n* is 21 in upper cluster, 16 in lower cluster. DLCO % predicted: *n* is 21 in upper cluster, 14 in lower cluster. T/S ratio is a measure for telomere length.

## Data Availability

Data can be requested from the corresponding author.

## Supplementary Materials

The following supporting information can be downloaded at https://www.mdpi.com/article/10.3390/ijms242316748/s1.
