# Supplementary material for: New Insights via RNA Profiling of Formalin-Fixed Paraffin-Embedded Lung Tissue of Pulmonary Fibrosis Patients
Source: Int J Mol Sci. 2023 Nov 25;24(23):16748. doi: 10.3390/ijms242316748 (PMC10706203; doi:10.3390/ijms242316748)
Supplement: Supplementary file 1 [file ijms-24-16748-s001.zip › ijms-2674627-supplementary.pdf]

Supplementary material

New insights via RNA profiling of formalin-fixed paraffin-embedded lung tissue of pulmonary fibrosis patients

Dymph Klay[1], Karin M Kazemier[2,3], Joanne J. van der Vis[1,4], Hidde M Smits[2], Jan C Grutters[1,3] and Coline HM van Moorsel[1*]

Table S1: List of primers used to determine RNA expression of genes encoding relevant proteins in lung tissue of patients with pulmonary fibrosis

Encoded protein	Gene name	Forward primer (5′-3′)	Reverse primer (5′-3′)
SP-B	*SFTPB*	TGAGGACATCGTCCACATCC	CCAGGAACTTCCTCATCGTGT
SP-C	*SFTPC*	AGCCAGAAACACACGGAGAT	GCCAGTGGAGCCGATG
SP-A2	*SFTPA2*	GGGACGGGAGAGATGGTG	CTCCAGGCAGCCCATTATTC
HIF1α	*HIF1A*	GTACCCTAACTAGCCGAGGAAGAA	GTGAATGTGGCCTGTGCAGT
HIF2α	*EPAS1*	GCGCTAGACTCCGAGAACAT	TGGCCACTTACTACCTGACCCTT
53BP1	*TP53BP1*	GTCAGGTCATTGAGCAGTTACCTC	TCCTCCACAGCAGGAGCAG
H2AFX	*H2AX*	ACTCAACTCGGCAATCCAAG	GGGTTAGCTGCAGAATTCCA
HOPX	*HOPX*	ACTTCAACAAGGTCGACAAGC	GGGTCTCCTCCTCGGAAA
CAV1	*CAV1*	CACATCTGGGCAGTTGTACC	CACAGACGGTGTGGACGTAG
CCSP	*SCGB1A1*	GAGCTTTCAGCGTGTCATCGAA	CCTGCCTCCCTCATGTCTTGAT
αSMA	*ACTA2*	CAAAGCCGGCCTTACAGAG	AGCCCAGCCAAGCACTG
VIM	*VIM*	TGTCCAAATCGATGTGGATGTTTC	TTGTACCATTCTTCTGCCTCCTG
TGFBR2	*TGFBR2*	ACGGTGCAGTCAAGTTTCCACAAC	ACACAGACTTCCTGTGGCTTCTCA
COL1A1	*COL1A1*	TACAGCGTCACTGTCGATGGC	TCAATCACTGTCTTGCCCCAG
COL1A2	*COL1A2*	CACCCAGAGTGGAGCAGTGG	TTCTTGGCTGGGATGTTTTCA
COL3A1	*COL3A1*	AATTTGGTGTGGACGTTGGC	TTGTCGGTCACTTGCACTGG
DDIT3	*DDIT3*	GGAGCATCAGTCCCCCACTT	TGTGGGATTGAGGGTCACATC
SMAD4	*SMAD4*	AAAACGGCCATCTTCAGCAC	AGGCCAGTAATGTCCGGGA
HSP90B1	*HSP90B1*	TTGCCAGACCATCCGTACTG	GAATTGGATGAAAGATAAAGCCCTTA
EDEM	*EDEM1*	CAAGTGTGGGTACGCCACG	AAAGAAGCTCTCCATCCGGTC
BiP	*HSPA5*	TGTTCAACCAATTATCAGCAAACTC	TTCTGCTGTATCCTCTTCACCAGT
XBP1	*XBP1*	TGGCCGGGTCTGCTGAGTCCG	ATCCATGGGGAGATGTTCTGG
ATF4	*ATF4*	GGGACAGATTGGATGTTGGAGA	ACCCAACAGGGCATCCAAGT
LC3B2	*MAP1LC3B*	AAACGCATTTGCCATCACA	GGACCTTCAGCAGTTTACAGTCAG
PSMD11	*PSMD11*	GCCATCTACTGCCCCCCTAA	ATGGATAATACCCGACTGCATGT
P16	*CDKN2A*	TGAGCTTTGGTTCTGCCATT	AGCTGTCGACTTCATGACAAG
TP53	*TP53*	TAACAGTTCCTGCATGGGCGGC	AGGACAGGCACAAACACGCACC
P21	*CDKN1A*	ACCTTCCAGCTCCTGTAACATACT	GTCTAGGTGGAGAAACGGGAA
ACTB	*ACTB*	CATTCCAAATATGAGATGCGTTGT	TGTGGACTTGGGAGAGGACT
RPL13A	*RPL13A*	CCTGGAGGAGAAGAGGAAAGAGA	TTGAGGACCTCTGTGTATTTGTCAA
EEF1A1	*EEF1A1*	CATCAAAGCAGTGGACAAGAAG	GGGTGGCAGGTATTAGGGATAA

Survival analysis of gene process groups with three or more genes

Extracellular matrix (ACTA2, VIM, COL1A1, SMAD4 and TGFBR2): p value: 0.18. Survival in patients with high RNA expression of 0-1, 2-3 and 4-5 extracellular matrix genes was 64, 22 and 36 months respectively.

Surfactant homeostasis (SFTPB, SFTPC and SFTPA2): p value: 0.34. Survival in patients with high RNA expression of 0-1 and 2-3 surfactant homeostasis genes was 64 and 29 months respectively.

DNA damage (53BP1, H2AX, TP53): p value: 0.58. Survival in patients with high RNA expression of 0-1 and 2-3 DNA damage genes was 36 and 31 months respectively.

Survival analysis of gene process groups with two genes

AEC1 involvement (CAV1 and HOPX): p value: 0.50. Survival in patients with high RNA expression of 0, 1 and 2 AEC1 involvement genes was 43, 38 and 23 months respectively.

Senescence (CDKN1A and CDKNA2A): p value: 0.01. Survival in patients with high RNA expression of 0, 1, 2 senescence genes was 93, 22 and 60 months respectively. This analysis is based on 30 out of 49 patients (61%).

Hypoxia (HIF1A and EPAS1): p value: 0.28. Survival in patients with high RNA expression of 0, 1, and 2 hypoxia genes was 38, 42, and 22 months respectively.
